# Imbalance of Lysine Acetylation Contributes to the Pathogenesis of Parkinson’s Disease

**DOI:** 10.3390/ijms21197182

**Published:** 2020-09-29

**Authors:** Rui Wang, Hongyang Sun, Guanghui Wang, Haigang Ren

**Affiliations:** Laboratory of Molecular Neuropathology, Jiangsu Key Laboratory of Neuropsychiatric Diseases & Department of Pharmacology, College of Pharmaceutical Sciences, Soochow University, Suzhou 215123, China; rwang0828@stu.suda.edu.cn (R.W.); 20184026011@stu.suda.edu.cn (H.S.)

**Keywords:** Parkinson’s disease, lysine acetylation, lysine acetyltransferases, lysine deacetylases, histone, nonhistone, mitochondria

## Abstract

Parkinson’s disease (PD) is one of the most common neurodegenerative disorders. The neuropathological features of PD are selective and progressive loss of dopaminergic neurons in the substantia nigra pars compacta, deficiencies in striatal dopamine levels, and the presence of intracellular Lewy bodies. Interactions among aging and genetic and environmental factors are considered to underlie the common etiology of PD, which involves multiple changes in cellular processes. Recent studies suggest that changes in lysine acetylation and deacetylation of many proteins, including histones and nonhistone proteins, might be tightly associated with PD pathogenesis. Here, we summarize the changes in lysine acetylation of both histones and nonhistone proteins, as well as the related lysine acetyltransferases (KATs) and lysine deacetylases (KDACs), in PD patients and various PD models. We discuss the potential roles and underlying mechanisms of these changes in PD and highlight that restoring the balance of lysine acetylation/deacetylation of histones and nonhistone proteins is critical for PD treatment. Finally, we discuss the advantages and disadvantages of different KAT/KDAC inhibitors or activators in the treatment of PD models and emphasize that SIRT1 and SIRT3 activators and SIRT2 inhibitors are the most promising effective therapeutics for PD.

## 1. Introduction

Parkinson’s disease (PD) is the second most common neurodegenerative disorder, affecting approximately 1% of the population over the age of 60 and, drastically, up to 5% of the population over 85 [1,2]. PD primarily causes motor symptoms such as bradykinesia, rigidity, resting tremor, and gait impairment, as well as some nonmotor symptoms, including olfactory disorders, sleep disturbances, anosmia, constipation, cognitive decline, and depression [3]. Selective and progressive loss of dopaminergic (DA) neurons in the substantia nigra pars compacta (SNpc) that leads to deficiencies in striatal dopamine production, as well as intracellular inclusions containing α-synuclein (α-syn) aggregates referred to as Lewy bodies (LBs) in surviving neurons, are the neuropathological features of PD [4,5]. Levodopa (L-DOPA) therapy to supplement dopamine is currently the most effective treatment for PD [6]. However, neither L-DOPA nor any other currently available therapies can slow down or prevent progressive DA neuronal degeneration in PD brains.

Although the pathogenesis of PD is still elusive, the interactions among aging and genetic and environmental factors are considered to underlie the common etiology of PD [7]. Aging is recognized as a critical risk factor for PD, and as the population ages, the incidence and prevalence of PD will likely increase by more than 30% by 2030 [8,9]. Although PD in most patients is idiopathic, approximately 5–10% of patients suffer from familial PD with Mendelian inheritance, and to date, more than 20 genes that cause familial PD have been identified; these genes are referred to as “PARK” genes, including *PARK1* (encoding α-syn), *PARK2* (encoding Parkin), *PARK6* (encoding PTEN-induced putative kinase 1, PINK1), *PARK8* (encoding leucine-rich repeat kinase 2, LRKK2), *PARK9* (encoding ATPase 13A2, ATP13A2), etc. [10,11]. Their main functions are shown in Table 1. In addition, genome-wide association studies (GWASs) have identified common genetic variants that increase PD risk [7,12]. In addition to aging and genetic factors, environmental factors are also closely related to PD occurrence and development. Environmental factors including the neurotoxin phenyl-1,2,3,6-tetrahydropyridine (MPTP) and its metabolite 1-methyl-4-phenylpyridinium iodide (MPP^+^); 6-hydroxydopamine (6-OHDA); paraquat; and several pesticides, such as rotenone and dieldrin, can induce PD or increase the risk of PD [7].

The molecular pathogenesis of PD is extremely complicated and involves changes in and dysregulation of various basic physiological processes, such as transcriptional changes, mitochondrial damage, metabolic dysfunction, protein clearance system impairment, oxidative stress, and neuroinflammation [13,14]. Research on genetic and environmental factors related to the neurodegeneration of DA neurons and α-syn aggregates has provided extensive evidence motivating us to deeply investigate PD pathogenesis. Recent investigations have suggested that lysine acetylation and deacetylation of numerous proteins, including histones and nonhistone proteins, as well as acetylation of α-syn itself, might be tightly associated with the molecular events underlying PD pathogenesis. In this review, we discuss the potential roles and mechanisms of lysine acetylation and deacetylation of histones and nonhistone proteins as well as the changes in lysine acetyltransferases (KATs) and lysine deacetylases (KDACs) associated with PD pathogenesis. In addition, we discuss promising KAT/KDAC inhibitors or activators for PD treatment.

## 2. Lysine Acetylation and Its Regulatory Mechanism and Functions

### 2.1. Lysine Acetylation and Its Functions

Lysine acetylation in histones was first described by Vincent Allfrey and his colleagues in 1964 [15]. Lysine acetylation is an evolutionarily conserved and reversible posttranslational modification (PTM) in eukaryotes that precisely governs protein functions and involves transfer of an acetyl group donated by acetyl coenzyme A (Ac-CoA) to the ε-amino side chain of a protein lysine residue. Lysine acetylation occurs in both histones and nonhistone proteins. Lysine acetylation of histones such as Histone 2A (H2A), Histone 2B (H2B), Histone 3 (H3), and Histone 4 (H4) generally results in transcriptional activation due to destabilization of DNA-histone binding, as acetylation of lysine neutralizes its positive charge, which prevents the formation of salt bridges with the negatively charged phosphate backbone of DNA [16]. In addition to histones, many nonhistone proteins in the cytoplasm and organelles are also dynamically acetylated and deacetylated; these changes are closely implicated in the regulation of various cellular processes, including gene transcription; cell cycle progression; DNA damage repair; cellular signal transduction; protein folding stability and aggregation; cytoskeleton organization; RNA processing and stability; and autophagy regulation [17,18].

**Figure 1:**
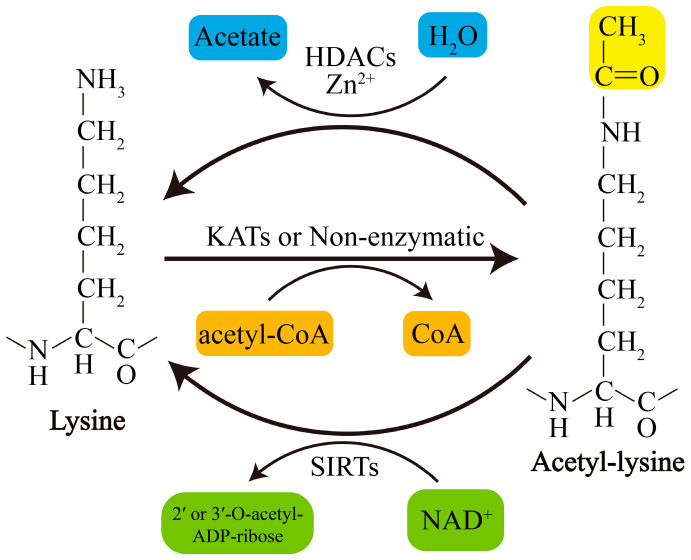
Schematic overview of lysine acetylation and deacetylation. Lysine acetylation, which is catalyzed by KATs, involves transfer of an acetyl group from Ac-CoA to the ε-amino side chain of lysine or occurs nonenzymatically. Deacetylation of lysine residues is catalyzed by Zn2^+^-dependent HDACs or by NAD^+^-dependent SIRTs. NAD^+^, nicotinamide adenine dinucleotide.

Acetylation of lysine residues can be catalyzed by KATs or modified by abundant Ac-CoA through nonenzymatic mechanisms. Conversely, lysine deacetylation is catalyzed by KDACs, which comprise two major groups with distinct catalytic mechanisms: NAD^+^-dependent Sirtuins (SIRTs) and Zn^2+^-dependent histone deacetylases (HDACs) (Figure 1). The acetylation levels of lysines are highly dynamic, and the balance between lysine acetylation and deacetylation is precisely controlled by KATs and KDACs as well as by the concentration of Ac-CoA in organellar compartments such as mitochondria [19,20].

### 2.2. KATs and KDACs in Humans and Their Involvement in PD

To date, at least 22 human KATs have been identified to display acetyltransferase activity; these KATs can be divided into three major families: the MYST family, the GNAT family, and the p300/CBP family [18,21] (Table 2). The substrate specificity of KATs is primarily determined by their subcellular distribution or interacting partners or by the availability of lysine in substrates [17]. Most KATs are localized mainly in the nucleus, where they mediate processes including but not limited to histone acetylation, and some KATs also located in the cytoplasm are responsible for cytoplasmic substrate acetylation [17]. Recently, GCN5-like 1 (GCN5L1) and Ac-CoA Acetyltransferase 1 (ACAT1) were identified as mitochondrial KATs that regulate mitochondrial functions by acetylating several mitochondrial substrates [22,23]. In addition, the well-known nuclear KAT8/MOF is also found to localize to mitochondria and affect mitochondrial functions [24]. The classifications, subcellular localization, involvement in PD models, and relevant substrates of these KATs are presented in Table 2. However, to date, only a small proportion of KATs have been identified to be related to PD (Table 2).

KDACs, originally referred to HDACs, were initially discovered to deacetylate histones in 1995 [32]. Later, they were also found to regulate nonhistone protein acetylation and cellular functions [33]. Currently, KDACs are grouped into two major types: NAD^+^-dependent SIRTs (SIRT1-7) and Zn^2+^-dependent HDACs (HDAC1-11). They can also be divided into four categories according to phylogeny and sequence similarities: Class I, Class IIa, Class IIb, and Class IV (Table 2). Recently, lymphoid enhancer-binding factor 1 (LEF1) and T cell-specific transcription factor 1 (TCF1) were identified as novel KDACs that are not related to the abovementioned types of KDACs [34]. Zn^2+^-dependent HDACs are primarily distributed in the nucleus or cytoplasm, although HDAC1 and HDAC7 are also found in mitochondria in some types of cells or under certain conditions [35,36]. In contrast, some SIRTs, including SIRT3-5, are restricted to the mitochondria, indicating their unique and crucial roles in mitochondria. However, it should be noted that several KDACs, such as that of SIRT4-6 and some class IIa HDACs, display weak or no deacetylase activity or target other types of acylation [17]. For example, SIRT5 exerts the activity of desuccinylase, demalonylase, and deglutarylase [37]; SIRT4 removes the acyl moieties from lysine residues such as methylglutaryl-, hydroxymethylglutaryl- and 3-methylglutaconyl-lysine [38]; SIRT6 functions to deacetylate long-chain fatty acyl groups rather than protein deacetylation [39]; The classifications, subcellular localization of KDACs, as well as their involvement in PD and the relevant acetylation of substrates are presented in Table 3.

Interestingly, the activity or expression levels of nuclear SIRT1 and mitochondrial SIRT3 are consistently decreased in PD tissues and different PD models. The activity or expression levels of nuclear HDAC2 and HDAC3 are increased in most PD models, but the expression levels of HDAC2 are decreased in tissues of PD patients. Furthermore, the activity or expression levels of two main cytoplasmic KDACs, HDAC6 and SIRT2, are downregulated and upregulated in most PD models, respectively (Table 3). Of note, beyond acetylation, several KATs/KDACs have activity of other acylation modifications including propionyl, butyryl, 2-hydroxyisobutyryl, crotonyl, malonyl, succinyl, or glutaryl modification. For example, p300 has crotonyltransferase activity [40], while KAT2A/GCN5 has both crotonyltransferase and uccinyltransferase activity [41,42], whereas HDAC1/2/3/8 and SIRT1-3 possess decrotonylating activity [43,44,45]. Whether these changes in KATs or KDACs in PD patients or models also cause variation of other acylation modifications, and the roles of these acylation variations in PD pathology, deserve further research.

## 3. Histone Acetylation in PD and Treatment Strategy Clues

### 3.1. Acetylation of Histones in Tissues of PD Patients

Histones are a major group of substrates for lysine acetylation, and histone acetylation is one of the main PTMs (also including histone phosphorylation, methylation, ubiquitination, etc.) that form the “histone code”; a hypothesis states that histone PTMs alone or in combination are believed to modulate chromatin structure and functions [72]. Histone acetylation or in combination with other PTMs can recruit or repel chromatin regulatory protein complexes to control gene expression or regulate other genomic functions, thereby involving various important cellular processes and disease progression [73]. Generally, acetylation of histones by KATs weakens their binding to DNA, relaxes chromatin and generally turns on gene transcription; in contrast, deacetylation of histones by HDACs results in condensation of chromatin and turns off gene transcription [74]. In addition, histone acetylation enables its interaction with bromodomain-containing proteins and transcriptional factors, thereby increasing the number of regulatory factors [75]. Hypoacetylated state of histones promotes their binding to transcriptional co-repressors, which are recruited by specific KDACs; not only that, KDACs can also inhibit the transcription initiation or are recruited to prepare for transcription repression [21].

Park et al. found that markedly higher lysine acetylation levels of specific sites in histones, including H2A, H2B, H3, and H4, are present in DA neurons from midbrain tissues of PD patients compared with matched controls [46]. The upregulation of histone acetylation is probably caused by the downregulation of KDACs, including HDAC1, HDAC2, HDAC4, HDAC6, and SIRT1, without significant changes in several KATs, including CBP, TIP60, and GCN5 [46]. Another study has indicated that increased H3 acetylation is mainly due to increased H3K14 and H3K18 acetylation in the postmortem PD primary motor cortex [76]. In addition, increases in histone acetylation are disease-dependently associated with PD progression [77].

Recently, a comparative study was conducted between two groups of fibroblasts from idiopathic PD and genetic PD*LRRK2* G2109S patients [48,78]. Mutations in the *LRRK2* gene, including G2109S and R1441G, account for an autosomal dominant type of familial PD [10]. Although the acetylation levels of total proteins in idiopathic PD fibroblasts compared with controls are increased, the acetylation levels of histones are decreased. Interestingly, the non-specific HDAC inhibitor trichostatin A (TSA) is harmful for idiopathic PD fibroblasts, while the histone acetyltransferase (HAT) inhibitor anacardic acid (AA) is beneficial [48]. The hyperacetylation of proteins in idiopathic PD cells may be due to mitophagy dysfunction and damaged mitochondrial accumulation that decreases NAD^+^ generation, which consequently reduces the activity of SIRTs. SIRT inhibition triggers an increase in the activity of class I and II KDACs, including HDAC2, HDAC3, and HDAC4, but decreases total HDAC activity, especially HDAC6 activity [48,78,79]. Therefore, HAT inhibitors or selective inhibitors targeting HDAC2, HDAC3, or HDAC4 are better than nonspecific HDAC inhibitors at restoring the balance of the acetylation levels of histones and reducing cell damage in PD.

There have been very large inconsistencies in histone acetylation and related enzyme changes in PD patient samples in these studies. The numbers of clinical samples may have been insufficient to reflect the actual changes in histone acetylation and KATs/KDACs. More clinical samples and more detailed mechanistic studies are needed in the future, especially studies in which specific KATs and KDACs are varied and the changes in histone acetylation are investigated in PD patients.

### 3.2. PD-Related Neurotoxins and Acetylation of Histones

H3 or H4 hyperacetylation is a key epigenetic change in DA neurons exposed to other PD-related neurotoxins, such as MPTP/MPP^+^, dieldrin, rotenone, and paraquat. For example, MPTP treatment in mice or MPP^+^ treatment in neuronal cells leads to increased lysine acetylation of histones, with corresponding decreases in HDAC1, HDAC2, HDAC4, HDAC6, and SIRT1 levels [46]. MPP^+^ treatment decreases SIRT1 expression and promotes acetylation of H3K14, which activates the hypoxia inducible factor 1α (*HIF-1α*) promoter to induce its transcription [59]. Consistent with the findings of this study, an increase in H3 acetylation in the striatum has also been found in mice treated with MPTP [80]. Pharmacological inhibition of HATs with the inhibitor garcinol suppresses MPP^+^-induced cell death, whereas HDAC1/2 genetic inhibition or treatment with the HDAC inhibitors MS-275 and TSA exacerbates cell damage [46]. However, another study has indicated that MPTP/MPP^+^ increases HDAC2 expression and that the HDAC1/2-specific inhibitor K560 attenuates MPTP/MPP^+^-induced neuronal cell death [47]. Interestingly, the pan-HDAC inhibitors TSA and hydroxamic acid (SAHA) do not inhibit MPP^+^-induced cell death, but valproate (VPA) exacerbates MPP^+^-induced cell death in vitro [47]. These results indicate that MPTP/MPP^+^ widely inhibits the expression of KDACs, and this inhibition may be accompanied by compensatory upregulation of individual HDACs under certain conditions.

Dieldrin, a toxin implicated in PD pathogenesis, induces H3 and H4 hyperacetylation in a time-dependent manner with CBP accumulation, and hyperacetylation modification is an early event in dieldrin-induced neurotoxicity [29]. Moreover, the HAT inhibitor AA dramatically attenuates dieldrin-mediated DA neuronal death [29]. Paraquat stimulates H3 rather than H4 hyperacetylation, which contributes to the death of DA neuronal cells, while paraquat-induced histone acetylation is related to decreases in the expression levels of HDAC4 and HDAC7 [51,52]. Rotenone increases H3K9 acetylation by reducing SIRT1 levels and thus activates p53 expression to promote neurodegeneration [60]. Resveratrol, a SIRT1 activator, attenuates rotenone-induced cell damage and p53 expression [60]. HAT inhibitors such as garcinol, AA, and curcumin reduce L-DOPA-induced dyskinesia in 6-OHDA-induced PD mouse models, suggesting that L-DOPA combined with a HAT inhibitor may have therapeutic potential for L-DOPA-induced dyskinesia in PD patients [81].

Manganese (Mn) neurotoxicity is tightly associated with Parkinson’s-like symptoms; however, unlike the above neurotoxins, MnCl_2_ significantly suppresses the acetylation of H3 and H4 in neuronal cells in a time-dependent manner while increasing the expression of HDAC3/4 and decreasing the expression of HAT [25]. In addition, the HAT inhibitor AA facilitates Mn-induced neuronal apoptosis, and HDAC inhibition by TSA has a neuroprotective effect [25]. Mn also induces a reduction in histone acetylation in astrocytes and inhibits the expression of astrocytic glutamate aspartate transporter (GLAST) and glutamate transporter 1 (GLT-1), which further contribute to neurotoxicity [82]. The HDAC inhibitor VPA reverses the Mn-induced reduction in histone acetylation, attenuates the Mn-induced decrease in astrocytic glutamate transporter expression, and reverses Mn-induced DA neurotoxicity [82].

It can be seen that several modulators of KATs or KDACs have inconsistent or even completely opposite effects in PD models. The reason may be that many HDAC and HAT modulators used in the above studies may have non-specific effects besides affecting enzyme activity and therefore have other effects on cellular function. For example, the HAT inhibitor garcinol also possesses antioxidant, anti-inflammatory, and neuroprotective properties, as well as inducing DNA damage and growth arrest in cancer cells [83,84]. Similarly, beyond inhibiting KAT activity, curcumin also has antioxidant, anti-inflammatory, and anti-tumor activity by affecting multiple enzymes [85]. Therefore, more specific inhibitors or activators targeting KATs or KDACs need to be selected to explore the role of histone acetylation in various PD models.

**Figure 2:**
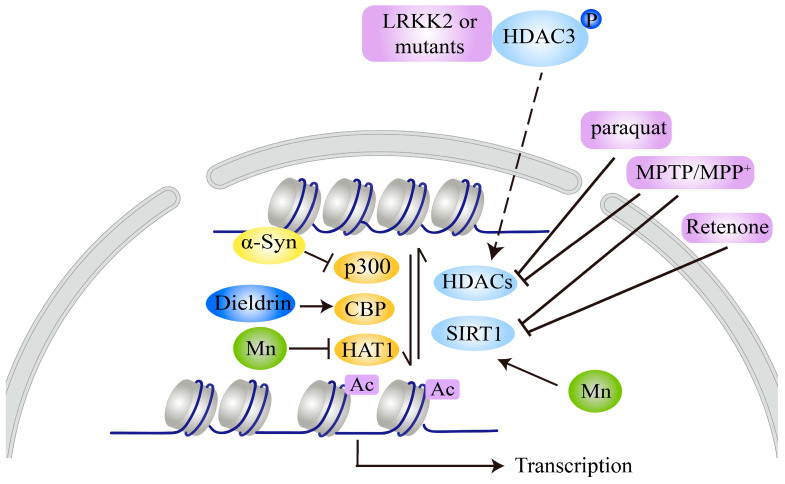
Lysine acetylation of histones mediated by PD-related neurotoxins and genetic factors. MPTP/MPP^+^, rotenone, paraquat, or dieldrin treatment leads to hyperacetylation of H3 and/or H4 by decreasing the activity or expression of certain HDACs or SIRT1 or by increasing CBP expression. α-syn causes hypoacetylation of H3 by binding to histones or inhibiting p300 expression and activity. LRKK2 or its mutants repress H4 acetylation by phosphorylating HDAC3 and promoting its nuclear translocation to increase its activity. Mn decreases H3 and H4 acetylation by increasing HDAC3/4 expression and decreasing HAT1 expression, respectively.

Nevertheless, the evidence indicates that histones are hypoacetylated or hyperacetylated in response to distinct PD-related neurotoxins. For example, MPTP/MPP^+^, dieldrin, paraquat, and rotenone all induce H3 or H4 hyperacetylation by decreasing the expression of KDACs, including several HDACs and SIRT1 (Figure 2), indicating that HAT inhibitors or SIRT1 activators are potential therapeutics for the balance of histone lysine acetylation and inhibition of neurotoxin-induced neurotoxicity. However, Mn leads to hypoacetylation of H3 and H4, suggesting that HDAC inhibitors may be valuable and effective treatment agents for Mn-induced PD neurotoxicity.

### 3.3. PD-Related Genes and Acetylation of Histones

Several products of PD-related genes have been shown to affect histone acetylation or to be regulated by histone acetylation [86]. α-syn plays vital roles in PD pathogenesis, as indicated by findings that α-syn aggregation in LBs in most PD cases and point mutations, duplications, or triplications of the *PARK1*/*SNCA* gene, which encodes α-syn, cause autosomal dominant familial forms of PD [87,88]. α-syn may cause neurotoxicity by interacting with histones to alter their acetylation. Binding of α-syn to histones in the nucleus of SNpc neurons promotes its fibrillation and toxicity [89]. Increased α-syn expression results in a reduction in H3 acetylation, and treatment with the HDAC inhibitor sodium butyrate (NaB) reverses α-syn-induced DNA damage [31]. Furthermore, α-syn reduces H3 acetylation, and the PD-related α-syn mutants A30P and A53T may aggravate this effect [90]. The HDAC inhibitors NaB and SAHA protect against α-syn-mediated neurotoxicity in both cellular and transgenic Drosophila models [90]. The HDAC inhibitor VPA has also been shown to protect against α-syn-mediated neurotoxicity in rat PD models [91]. Of note, there is no direct association between α-syn and H3, indicating that the reduction in histone acetylation caused by α-syn may occur through histone masking [92]. Through a similar mechanism, nuclear α-syn binds to the gene promoter region of peroxisome proliferator-activated receptor γ coactivator-1α (PGC-1α), a mitochondrial transcription factor whose dysfunction contributes to mitochondrial dysfunction and oxidative stress in PD pathogenesis; prevents histone acetylation; and represses PGC-1α expression [93]. α-syn also decreases histone acetylation, probably by reducing p300 levels and its HAT activity [30].

LRRK2 phosphorylates HDAC3 at S424 and stimulates its deacetylase activity through an interaction, subsequently promoting deacetylation of H4K5/H4K12 and leading to repression of gene transcription. LRRK2 also promotes the nuclear translocation of HDAC3 by phosphorylating karyopherin subunits α2 and α6 [49]. The phosphorylation and nuclear translocation of HDAC3 are further increased under 6-OHDA treatment, and LRRK2 ultimately promotes 6-OHDA-induced damage by positively modulating HDAC3 [49]. The HDAC inhibitor VPA improves motor and nonmotor behaviors in PD mice harboring the LRRK2 R1441G mutation. VPA administration also increases the levels of histone acetylation and the numbers of DA neurons in the SNpc in transgenic mice [94].

Interestingly, in contrast to MPTP/MPP^+^, dieldrin, paraquat, and rotenone, and similar to Mn, α-syn and LRRK2 lead to hypoacetylation of H3 or H4. α-syn inhibits histone acetylation by binding to histones or decreasing p300 expression, while LRKK2 and its pathogenic mutants reduce histone acetylation by promoting HDAC3 phosphorylation and nuclear translocation to increase its deacetylase activity (Figure 2), suggesting that HDAC inhibitors may be valuable and effective for α-syn-, LRRK2-, or Mn-induced PD neurotoxicity. However, whether other genetic factors have effects on histone acetylation still lacks experimental evidence. These pathogenic factors, such as genetic factors (α-syn and LRRK2) and environmental toxins, have similar or different effects on histone acetylation, which may be related to their involvement in general or specific cellular signals/functions in nerve cells [10,95]. Recently, Mn has been shown to promote α-syn aggregation and transmission [96], which may contribute to hypoacetylation of histones, similar to the direct effect of α-syn on histone acetylation. More detailed studies are needed to clarify the mechanisms for these differences, which will promote the discovery of the role of histone acetylation in PD pathology and be beneficial for choosing appropriate KAT or KDAC modulators for treatment of different PD models.

Furthermore, α-syn expression may also be regulated by histone acetylation. Voutsinas et al. found that epigenetic silencing of the A53T mutant allele involves histone deacetylation modification, as the use of an HDAC inhibitor can reactivate allele expression [97]. Consistent with this finding, H3K27 acetylation-enriched enhancer regions have been identified at the *SNCA* gene locus [98]. These results indicate that α-syn expression may also be regulated by histone acetylation. MPTP increases the levels of α-syn, which is involved in the activation of proteinase-activated receptor 2/NF-κB signaling and H3 acetylation in the *α-syn* gene promoter region [99].

## 4. Lysine Acetylation of Nonhistone Proteins in PD Pathogenesis

In addition to changes in acetylation of histones, accumulating evidence suggests that imbalance between acetylation and deacetylation modifications on nonhistone proteins is also associated with the pathogenesis of PD.

### 4.1. PD-Related Genes and Nonhistone Acetylation

**Figure 3:**
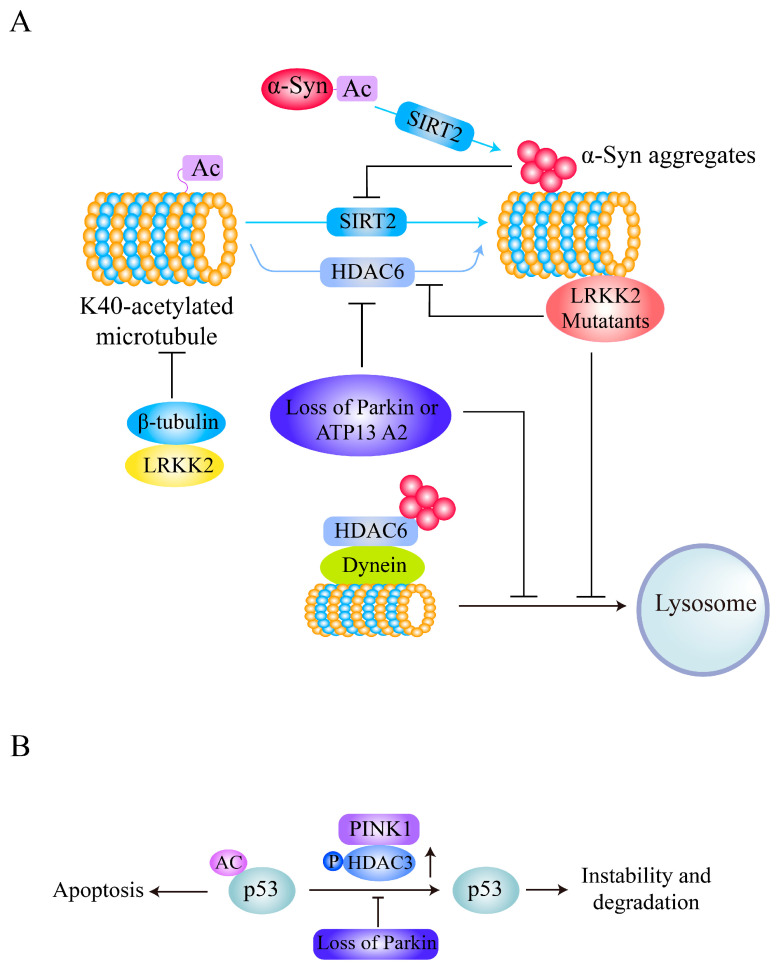
Acetylation of α-tubulin or p53 is affected by PD-related genetic factors. (**A**) Deacetylation of α-syn by SIRT2 facilitates its aggregation and toxicity. α-syn activates SIRT2 to inhibit acetylation of α-tubulin, and the deacetylated α-tubulin associates with α-syn oligomers to form a toxic complex. LRRK2 binds to β-tubulins and inhibits α-tubulin acetylation, and LRKK2 mutants preferentially bind to deacetylated MTs and disrupt axonal transport. However, loss of Parkin or ATP13A2 leads to repression of HDAC6, hyperacetylation of α-tubulin, and dysfunction of aggregate degradation. (**B**) PINK1 phosphorylates HDAC3 to increase its p53-deacetylating activity, and deacetylated p53 is unstable. Loss of PINK1 leads to hyperacetylation of p53 and thus induces neuronal cell damage.

Interestingly, α-syn itself can be acetylated and deacetylated, and its deacetylation status mediates its neurotoxicity. In addition to N-terminal acetylation of α-syn, an irreversible modification, lysine acetylation of α-syn is an important mechanism for regulation of its aggregation and neurotoxicity [100,101,102,103]. α-syn can be acetylated at K6 and K10 to inhibit its aggregation and toxicity, and these modifications are reversed by SIRT2 (Figure 3A). SIRT2 deletion promotes α-syn acetylation and protects against α-syn- or MPTP-induced neuronal toxicity in vivo [64]. Treatment with the SIRT2 inhibitor AGK2 and genetic inhibition of AK-1 or SIRT2 also alleviates α-syn-mediated DA neuronal loss both in vitro and in a Drosophila PD model [104].

Furthermore, α-syn also has an inhibitory effect on the acetylation of α-tubulin via a SIRT2-related mechanism, impairing microtubule (MT) stability and contributing to PD pathology (Figure 3A), and the SIRT2 inhibitor AK-1 partially reverses the reduction in acetylation of α-tubulin mediated by α-syn and restores MT stability [65]. In addition, deacetylated α-tubulin interacts with α-syn oligomers to form a toxic complex [105]. Although HDAC6 also deacetylates α-tubulin and HDAC6 inhibition protects DA neurons from α-syn toxicity in a rat model of PD [106], depletion of HDAC6 significantly enhances α-syn-induced DA neuron loss and locomotor dysfunction in PD models [107,108]. HDAC6 also increases in expression and colocalizes with α-syn in the perinuclear region to form aggresome-like bodies and inhibit its neurotoxicity through a mechanism involving the aggresome-autophagy pathway [109]. These results suggest that SIRT2-mediated α-tubulin deacetylation plays essential roles in α-syn-mediated PD pathogenesis and that targeting SIRT2 inhibition rather than HDAC6 inhibition is a potential therapeutic strategy for α-syn-related PD.

LRRK2 directly interacts with β-tubulins and has an inhibitory effect on K40 acetylation of α-tubulin and a dramatic positive effect on α-tubulin acetylation in mouse embryonic fibroblasts derived from *LRRK2*-knockout mice [28]. Interestingly, LRRK2 mutants have been found to preferentially bind to deacetylated MTs, disrupt axonal transport in neurons, and cause locomotor deficits in a PD Drosophila model (Figure 3A) [27]. Increases in MT acetylation caused by the α-tubulin acetyltransferase αTAT1 or deacetylase inhibitors prevent the interaction between LRRK2 mutants and MTs, and genetic inhibition of HDAC6 or SIRT2 or administration of the SIRT2 inhibitor AGK2 reverses the defective axonal transport and locomotor behavior induced by LRRK2 mutants [27], indicating that deacetylase inhibitors targeting HDAC6 or SIRT2 are potential therapeutics for axonal transport dysfunction in mutant LRRK2-related PD.

Loss of Parkin (encoded by the *PARK2* gene mutations which cause early-onset autosomal recessive familial PD) induces hyperacetylation of MTs in both DA neuron cell bodies and fibers and disrupts MT stability [53]. On the other hand, deacetylase activity of HDAC6 directed toward α-tubulin is required for Parkin movement and accumulation in the centrosome [110] and promotes mitophagy [111]. Similarly, cells and *Drosophila melanogaster* and mouse tissues with loss of ATP13A2, encoded by the *PARK9* gene (mutations in which are also related to the early-onset autosomal recessive form of familial PD) exhibit increased acetylation of α-tubulin and cortactin and impaired autophagosome-lysosome fusion, which are associated with reduced lysosomal localization and activity of HDAC6 (Figure 3A). In addition, wild-type HDAC6, but not a deacetylase-inactive mutant, antagonizes hyperacetylation and restores autophagosome-lysosome fusion [54], indicating that reduced activity of HDAC6 deacetylase is an essential pathological factor related to impaired autophagy flux in PD pathogenesis. Activation or overexpression of HDAC6 is thus a potential therapeutic strategy for Parkin- or ATP13A2-related PD.

Another PD-related protein, PINK1, is encoded by the *PINK1*/*PARK6* gene, mutations in which cause autosomal recessive early-onset PD [112,113]; this protein has been shown to bind to HDAC3 and increase its histone deacetylase activity via phosphorylation in neuronal cells [50]. HDAC3 phosphorylated by PINK1 binds to p53, decreases p53 acetylation and stability, and thus inhibits p53-mediated neuronal cell damage (Figure 3B), and HDAC3 deficiency abolishes the effect of PINK1 on p53 [50]. Interestingly, PD-related PINK1 mutations cause PINK1 to lose the ability to influence HDAC3 deacetylase activity and increase susceptibility to p53-mediated neurodegeneration [50].

Therefore, changes in α-tubulin acetylation are common pathologies mediated by PD-related genetic factors. α-tubulin acetylation is a reversible modification affecting cytoskeletal organization and can be reversed by HDAC6 or SIRT2 [17,114,115]. PD-related gene products such as α-syn, LRKK2, Parkin, and ATP13A all have inhibitory effects on K40 acetylation of α-tubulin (Figure 3). However, the underlying mechanisms of treatment strategies targeting abnormal tubulin acetylation caused by these gene products are very different. Considering the gain-of-function mutations of α-syn and LRKK2 in PD and the contribution of deacetylated α-tubulin to their neurotoxicity, as well as the role of HDAC6 in protein aggregate degradation and autophagy [116], targeting SIRT2 inhibition may be a valuable potential therapeutic strategy for α-syn- or LRKK2-related PD. In addition, HDAC6 activation is a potential therapeutic strategy for PD induced by loss of function of Parkin or by ATP13A2 mutation.

### 4.2. PD-Related Neurotoxins and Nonhistone Acetylation

**Figure 4:**
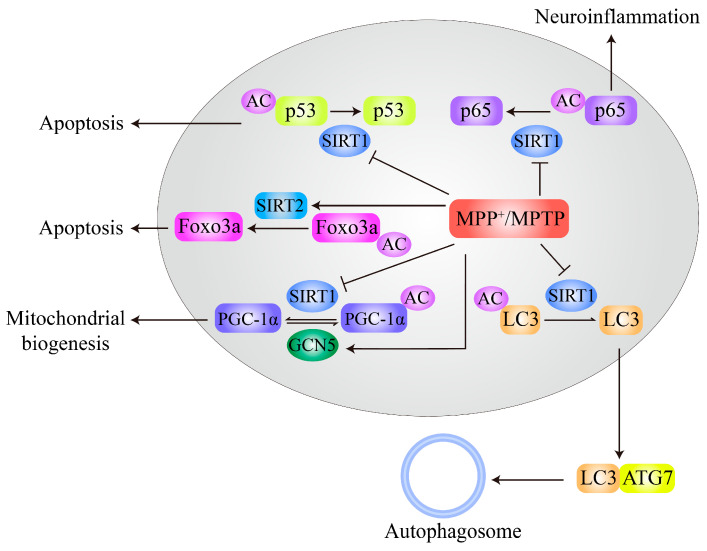
Effects of MPTP/MPP^+^ on acetylation of nonhistone proteins. MPTP/MPP+ inhibits SIRT1 expression or activity to block deacetylation of various substrates, such as p53, p65, PGC-1α, and LC3, which leads to neuronal apoptosis, neuroinflammatory activation, mitochondrial dysfunction, and autophagy inhibition, respectively. However, MPTP/MPP+ also promotes GCN5 expression to increase PGC-1α acetylation. In addition, MPTP/MPP^+^ activates SIRT2 to induce deacetylation of Foxo3a, enhancing Bim transcription and thus leading to neurodegeneration.

MPTP treatment decreases SIRT1 expression and subsequently increases acetylation of MT-associated protein 1 light chain 3 (LC3), thus inhibiting autophagic degradation of α-syn and leading to neurodegeneration of DA neurons (Figure 4). Resveratrol activates SIRT1 and deacetylates LC3 to relocalize it to the cytoplasm for α-syn degradation by autophagy [57]. MPTP administration in mice also increases S-nitrosylation of SIRT1 and leads to inhibition of its deacetylase activity, subsequently resulting in hyperacetylation and activation of p53 and NF-κB subunit p65. Activation of p53 and p65 promotes neuronal apoptosis and increases glial proinflammatory gene expression, respectively (Figure 4), both of which are related to PD pathogenesis [58]. Deacetylation of Foxo3a by SIRT2 is stimulated by MPTP or MPP^+^ treatment and increases Bim expression, thus contributing to MPTP/MPP^+^-induced neuronal apoptosis in PD models. In addition, neurodegeneration induced by MPTP can be prevented by SIRT2 inhibition [66] as well as by SIRT1 overexpression [56].

In addition, PGC-1α, which plays essential roles in mitochondrial biogenesis, metabolism, and oxidative stress, has been widely implicated in PD pathogenesis [117]. PGC-1α is directly acetylated by GCN5 or p300, and its acetylation results in inhibition of PGC-1α transcriptional activity [118]. Increases in GCN5-mediated PGC-1α acetylation contribute to MPP^+^-induced neuronal toxicity (Figure 4) [26]. Interestingly, SIRT1 also directly deacetylates PGC-1α and enhances its functions [119]. Therapeutic reagents that inhibit GCN5 or p300 or activate SIRT1 modification may have potential value for PD treatment.

Furthermore, 6-OHDA treatment in cells and rats also decreases the levels of SIRT1 and results in an increased ratio of acetylated BMAL1, thus affecting the expression of circadian genes and increasing oxidative stress. An activator of SIRT1, resveratrol, decreases BMAL1 acetylation and inhibits its interaction with cryptochrome 1, thereby alleviating the impairment of antioxidant activity induced by 6-OHDA [62]. Moreover, 6-OHDA treatment significantly increases HDAC6 expression and decreases the acetylation levels of peroxiredoxin1/2, and inhibition of HDAC6 with the specific inhibitor tubastatin A increases acetylation of peroxiredoxin1/2, reduces ROS production, and ameliorates 6-OHDA neurotoxicity [55]. Pharmacological inhibition of HDAC6 with tubastatin A also attenuates NLRP3 inflammation in glial cells and inhibits DA neuronal degeneration while restoring the acetylation levels of peroxiredoxin2 [120]. In addition, a p300/CBP activator, CTPB, promotes the survival and neurite growth of neuronal cells and protects cells from cell death induced by 6-OHDA [121]. However, one study has indicated that 6-OHDA reduces SIRT2 activity by decreasing NAD^+^ levels and increasing α-tubulin acetylation and that overexpression of HDAC6 and SIRT2 reverses the changes in tubulin acetylation and MT dynamics in 6-OHDA-treated cells [67].

Together, these findings suggest that decreased activity or expression of SIRT1 is closely related to PD pathogenesis in MPTP/MPP^+^- and 6-OHDA-induced PD models (Figure 4) and PD patients [46,56]. Interestingly, SIRT1 activation not only restores the balance of acetylation of nonhistone proteins but also reverses histone hyperacetylation-induced neuronal toxicity caused by MPTP/MPP^+^ and rotenone. However, the correlations between PD genetic factors and SIRT1 activity or expression are still unclear.

### 4.3. Acetylation of Mitochondrial Proteins in PD

Mitochondrial dysfunction is one of the central pathogenic mechanisms of DA neuron degeneration and PD pathogenesis [4,122]. Mitochondrial functions are highly regulated by the dynamic acetylation modification of mitochondrial proteins [123]. With the development of acetylation proteomics techniques in recent years, it has been found that more than 60% of mitochondrial proteins contain acetylation modification sites [123]. Although mitochondrial protein acetylation is commonly recognized as nonenzymatic, mitochondrial KATs, such as ACAT1 and GCN5L1, also participate in mitochondrial protein acetylation [22,23]. However, deacetylation of mitochondrial proteins is performed mainly by SIRT3 [123,124].

**Figure 5:**
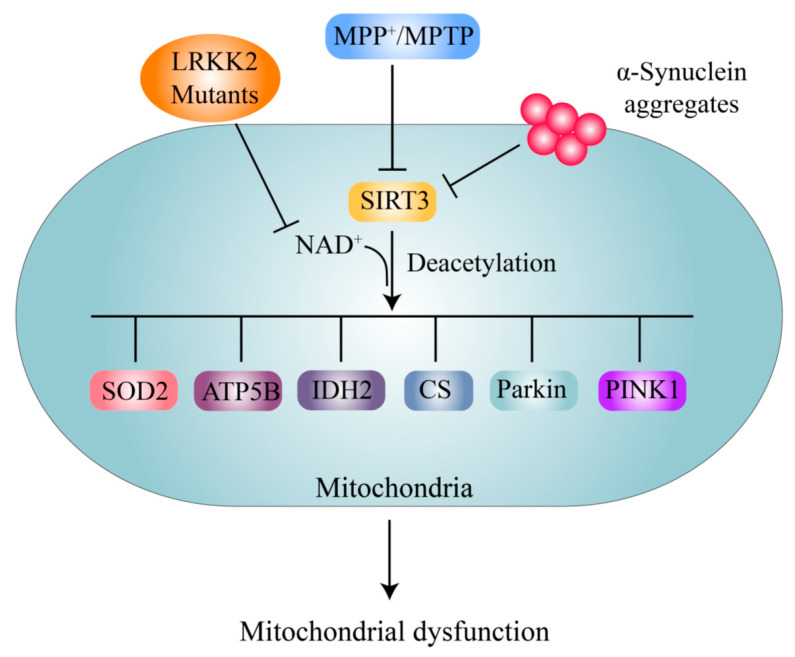
Acetylation of mitochondrial proteins by PD-related neurotoxins or genetic factors. α-syn and MPTP/MPP^+^ reduce SIRT3 expression, while LRKK2 G2109S inhibits SIRT3 activity by decreasing NAD^+^ levels to prevent deacetylation of mitochondrial substrates, including SOD2, ATP5B, GS, and IDH2, thereby reducing their activity decline and leading to mitochondrial dysfunction.

Notably, neurons treated with MPTP exhibited decreased levels of the SIRT3 protein, which is responsible for the high levels of acetylation of superoxide dismutase 2 (SOD2) at the K130 site and ATP synthase β at the K485 site, as well as loss of DA neurons (Figure 5). SIRT3 overexpression protects against and SIRT3 knockout promotes MPTP-induced DA neuronal loss [69]. MPP^+^ treatment also decreases SIRT3 expression and leads to increased acetylation of citrate synthase (CS) and isocitrate dehydrogenase 2 (IDH2) and decreased enzymatic activity of these proteins (Figure 5) [70]. SIRT3 deacetylates SOD2 at the K68 site and increases its ROS-eliminating activity, thus repressing DA neuronal degeneration in PD [68]. Lysine acetylation changes are detectable in the mitochondria in A53T-α-syn/*PINK*^-/-^ mice compared with wild-type controls, and SIRT3 levels are significantly decreased [125]. Furthermore, decreased expression or activity of SIRT3 contributing to increased acetylation of SOD2 is associated with genetic factors, including α-syn and *LRRK2* G2019S [63,71]. Interestingly, SIRT3 binds directly to the PD genetic factors PINK1 and Parkin and promotes their deacetylation and thus angiogenesis, and SIRT3 inhibition leads to hyperacetylation of PINK1/Parkin and impairment of mitophagy under stress [126]. However, whether the hyperacetylation of PINK1/Parkin participates directly in PD pathogenesis remains unclear. It has also been suggested that SIRT5 protects against motor deficits and DA neuron degeneration in MPTP-treated mice [127]. However, whether SIRT5 exerts a DA neuron protective function depends on its deacetylase activity, and the mechanism, is not yet clear.

SIRT3 plays essential roles in regulating mitochondrial functions, such as ATP generation, energy metabolism, antioxidant stress, survival, and proliferation, by deacetylating various substrates, such as long-chain acyl-CoA dehydrogenase (LCAD), ATP synthases, NADH dehydrogenase (NDUFA9), the mitochondrial protease Lon peptidase 1 (LONP1), and glycogen synthase kinase 3 beta (GSK3β) [128]. Although these proteins have been implicated to play roles in PD, whether acetylation of these proteins is directly involved in PD pathogenesis still lacks relevant research. In addition, whether mitochondrial KATs, ACAT1, and GCN5L1 participate in the acetylation modification of these proteins and their roles in PD pathogenesis needs further investigation.

Although further research on SIRT3 and its substrates in the context of PD needs to be performed, activation of SIRT3 has been shown to be a very promising treatment strategy for PD. Overexpression of SIRT3 or enhancement of SIRT3 activity by NAD^+^ precursors has been found to protect DA neurons in various PD models [68,69,129,130]. Recently, human clinical trials have indicated that supplementation with the NAD^+^ precursor nicotinamide riboside (NR) is well tolerated, elevates NAD^+^ levels in healthy adults, and reduces the levels of circulating inflammatory cytokines in patients [131,132]. Another NAD^+^ precursor, nicotinamide mononucleotide (NMN), also increases NAD^+^ levels in mitochondria and protects mitochondria [133]. These findings suggest that NAD^+^ precursors are potential clinical drugs for PD treatment.

## 5. Conclusions and Perspectives

Abnormal and imbalanced acetylation of histones as well as nonhistone proteins is tightly associated with many human diseases, including PD; therefore, KATs and KDACs, which directly participate in acetylation or deacetylation modification, are attractive therapeutic targets in PD treatment. Activators of SIRT1 and SIRT3 and inhibitors of SIRT2 are considered to be the most promising representative agents for the treatment of PD. Although the effects of nonspecific HDAC inhibitors are still controversial in different PD models or even in PD models of the same type, it should be noted that certain KAT inhibitors and specific HDAC inhibitors may be effective for several PD models.

Altered acetylation of histones, especially H3 and H4, has been widely studied in the context of PD. Although increased levels of histone acetylation are found in the brains of PD patients [46], histones can be either hypoacetylated or hyperacetylated in different PD models (Figure 2). Most PD-related neurotoxins, such as MPTP/MPP^+^, rotenone, paraquat, and dieldrin, can induce H3 or H4 hyperacetylation. However, genetic factors such as α-syn and LRRK2, as well as the neurotoxin Mn, can lead to hypoacetylation of histones. It is not yet clear whether altered histone acetylation is a causative factor of DA neurodegeneration or initiates a compensatory response to reduce DA neurodegeneration. Nevertheless, numerous studies have suggested that HAT inhibitors, HDAC inhibitors, or SIRT1 activators targeting histone acetylation balance have potential therapeutic value for various PD models. However, more selective inhibitors or activators targeting different PD models need to be selected.

Alterations in the acetylation levels of nonhistone proteins, mainly α-tubulin at K40, contribute to the pathogenesis of PD caused by genetic factors such as α-syn, LRRK2, Parkin, and ATP13A2 [27,28,53,54,65]. These alterations mainly involve changes in the activity or protein levels of SIRT2 and HDAC6, two cytoplasmic KDACs (Figure 3A). Interestingly, the acetylation of α-syn itself can inhibit its neurotoxicity, and deacetylation by SIRT2 enhances its neurotoxicity [64]. Inhibition of SIRT2 activity is the most promising therapeutic strategy for the treatment of α-syn-mediated neurodegeneration. However, targeting HDAC6 for PD treatment is still controversial [116]. PD-related neurotoxins cause changes in the acetylation levels of a variety of nonhistone proteins, involving alterations in signaling pathways such as autophagy, apoptosis, neuroinflammation, oxidative stress, and mitochondrial biogenesis pathways, mainly by inhibiting SIRT1 expression or activity (Figure 4). Interestingly, it can be concluded that SIRT1 activation exerts a general alleviating effect on DA neurodegeneration by targeting not only nonhistone acetylation balance but also histone acetylation balance (Figure 2 and Figure 4). The changes in the acetylation levels of mitochondrial proteins in PD models are mainly mediated by decreased activity or protein levels of SIRT3 (Figure 5), and activation of SIRT3 is the most attractive and valuable therapeutic strategy for PD treatment.

Thus far, research on the mechanisms of changes in the acetylation levels of proteins, especially histones, that occur in PD has been very limited. More systematic and in-depth studies are needed to explore the specific KATs or KDACs related to PD pathology and their underlying mechanisms. There is also a need to clarify why changes in protein acetylation levels and related KATs or KDACs in different PD models are different or even opposite. More importantly, for preclinical research and clinical trials on PD, more specific and less toxic inhibitors or activators for KATs and KDACs must be developed.

## Figures and Tables

**Table 1:** PD-Causing Genes and their Main Functions.

Gene	Encoding Protein	Main Functions
*PARK1/SNCA*	α-syn	Synaptic function
*PARK2/PRKN*	Parkin	Ubiquitination; synaptic function
*PARK3/PARK3*	Parkinson disease 3	Unknown
*PARK5/UCHL1*	Ubiquitin C-terminal hydrolase L1	Ubiquitin-proteasome system; autophagy
*PARK6/PINK1*	PINK1	Mitochondrial function; mitophagy
*PARK7/DJ-1*	DJ-1	Mitochondrial function; anti-oxidative stress;
*PARK8/LRKK2*	LRKK2	Vesicle transport; autophagy; synaptic function
*PARK9/ATP13A2*	ATP13A2	Lysosomal function; mitochondrial function
*PARK10/PARK10*	Parkinson disease 10	Unknown
*PARK11/GIGYF2*	GRB10 interacting GYF protein 2	Growth factors (IGFs) signaling
*PARK12/PARK12*	Parkinson disease 12	Unknown
*PARK13/HTRA2*	HtrA serine peptidase 2	Mitochondrial function; anti-oxidative stress
*PARK14/PLA2G6*	Phospholipase A2 group VI	Lipid metabolism; mitochondrial function
*PARK15/FBXO7*	F-box protein 7	Mitochondrial function; ubiquitination
*PARK16/PARK16*	Parkinson disease 16	Unknown
*PARK17/VPS35*	VPS35 endosomal protein sorting factor	Vesicle transport
*PARK18/EIF4G1*	Eukaryotic translation initiation factor	Translation initiation
*PARK19/DNAJC6*	DnaJ heat shock protein family (Hsp40) member C6	Endocytosis; synaptic function
*PARK20/SYNJ1*	Synaptojanin 1	Endocytosis; synaptic function
*PARK21/TMEM230*	Transmembrane protein 230	Vesicle trafficking; synaptic function
*PARK22/CHCHD2*	Coiled-coil-helix-coiled-coil-helix domain containing 2	Mitochondrial function
*PARK23/VPS13C*	Vacuolar protein sorting 13 homolog C	Mitochondrial function; mitophagy

**Table 2:** Human KATs and their Involvement in PD.

Family	Name	Subcellular Localization	PD Model/KAT Change/Substrate Acetylation Changes
GNAT	KAT1/HAT1	Nucleus	Mn/expression ↓/H3 and H4 ↓ [25].
GNAT	KAT2A/GCN5	Nucleus	MPP^+^/activity ↑/PGC-1α ↑ [26].
GNAT	KAT2B/PCAF	Nucleus	NA
GNAT	KAT9/ELP3	Nucleus/cytoplasm	NA
GNAT	αTAT1/ATAT1	Cytoplasm/membrane	LRRK2 knockout/NA/α-tubulin ↑; LRKK2 R1441C or Y1699C/NA/α-tubulin ↓ [27,28].
p300/CBP	KAT3A/CBP	Nucleus/cytoplasm	Dieldrin/expression ↑/H3 and H4 ↑ [29].
p300/CBP	KAT3B/p300	Nucleus/cytoplasm	α-syn/expression and activity ↓/NF-κB-p65 or H3 ↓ [30,31].
MYST	KAT5/TIP60/PLIP	Nucleus/cytoplasm	NA
MYST	KAT6A/MOZ/MYST3	Nucleus	NA
MYST	KAT6A/MORF/MYST4	Nucleus	NA
MYST	KAT7/HBO1/MYST2	Nucleus	NA
MYST	KAT8/MOF/MYST1	Nucleus/mitochondria	NA
Other	KAT4/TAF1/TAFII250	Nucleus	NA
Other	KAT12/TFIIC90	Nucleus	NA
Other	KAT13A/SRC-1/NCOA1	Nucleus	NA
Other	KAT13B/SRC-3/NCOA3	Nucleus/cytoplasm	NA
Other	KAT13C/SRC-2/NCOA2	Nucleus	NA
Other	KAT13D/CLOCK	Nucleus/cytoplasm	NA
Other	ATF-2/CREB2	Nucleus/cytoplasm	NA
Other	NAT10	Nucleus	NA
Other	ACAT1	Mitochondria	NA
Other	GCN5L1	Mitochondria	NA

↑, upregulation; ↓, downregulated; NA, not available; PGC-1α, peroxisome proliferator-activated receptor γ coactivator-1α; NF-κB, nuclear factor Kappa-B.

**Table 3:** Human KDACs and their Involvement in PD.

Class	Name	Subcellular Localization	PD Model/KDAC Change/Substrate Acetylation Level Change
I	HDAC1	Nucleus	Patient tissues, MPTP or MPP^+^/expression ↓/H2A, H2B, H3 and H4 ↑ [46].
I	HDAC2	Nucleus	Patient tissues, MPTP or MPP^+^/expression ↓/H2A, H2B, H3 and H4 ↑ [46]; MPP^+^/expression ↑/NA [47]; idiopathic PD fibroblasts/expression ↑/H3 ↓; *LRRK2* G2109S PD fibroblasts/expression ↑/H3 ↓ [48].
I	HDAC3	Nucleus	Idiopathic PD fibroblasts/expression ↑/H3 ↓ [48]; Mn/expression ↑/H3 and H4 ↓ [25]; LRRK2 or mutation/phosphorylation ↑, nuclear translocation ↑ and activity ↑/H4 ↓ [49]; PINK1 mutation/phosphorylation ↓ and activity ↓/p53 ↑ [50].
I	HDAC8	Nucleus/cytoplasm	NA
IIa	HDAC4	Nucleus/cytoplasm	Patient tissues, MPTP or MPP^+^/expression ↓/H2A, H2B, H3 and H4 ↑ [46]; paraquat/expression ↓/H3 ↑ [51,52]; idiopathic PD fibroblasts/expression ↑/H3 ↓, *LRRK2* G2109S PD fibroblasts/expression ↑/H3 ↓ [48]; Mn/expression ↑/H3 and H4 ↓ [25].
IIa	HDAC5	Nucleus/cytoplasm	NA
IIa	HDAC7	Nucleus/cytoplasm	Paraquat/expression ↓/H3 ↑ [51,52].
IIa	HDAC9	Nucleus/cytoplasm	NA
IIb	HDAC6	Primarily cytoplasm	Patient tissues, MPTP or MPP^+^/expression ↓ [46]; idiopathic PD fibroblasts/expression ↓, *LRRK2* G2109S PD fibroblasts/expression ↓ [48]; Parkin absence/NA/α-tubulin ↑ [53]; ATP13A absence/activity ↓/α-tubulin ↑ [54]; 6-OHDA/expression ↑/peroxiredoxin 1/2 ↓ [55].
IIb	HDAC10	Primarily cytoplasm	NA
III	SIRT1	Nucleus	Patient tissues, MPTP or MPP^+^/expression ↓/H2A, H2B, H3 and H4 ↑ [46]; patient tissues/activity ↓ [56]; MPTP/expression ↓/LC3 ↑ [57]; MPTP/S-nitrosylation ↑ and activity ↓/p53 and NFκB-p65 ↑ [58]; MPP^+^/expression ↓/H3 and PGC-1α ↑ [59]; rotenone/expression ↓/H3 ↑ [60,61]; 6-OHDA/expression ↓/BMAL1 ↑ [62]; *LRRK2* G2019S iPSC-derived dopaminergic cultures/activity ↓/p53 ↑ [63].
III	SIRT2	Cytoplasm	MPTP/activity ↑/α-syn ↓ [64]; α-syn/activity ↑/α-tubulin ↓ [65]; MPTP or MPP^+^/activity ↑/Foxo3a ↓ [66]; 6-OHDA/activity ↓/α-tubulin ↑ [67].
III	SIRT3	Mitochondria	Patients/NA/MnSOD ↑ [68]; MPTP/expression ↓/SOD2 and ATP5B ↑ [69]; MPP^+^/expression ↓/citrate synthase and isocitrate dehydrogenase 2 ↑ [70]; α-syn/expression ↓/SOD2 ↑ [71]; *LRRK2* G2019S iPSC-derived dopaminergic cultures/activity ↓/SOD2 ↑ [63].
III	SIRT4	Mitochondria	NA
III	SIRT5	Mitochondria	NA
III	SIRT6	Nucleus	NA
III	SIRT7	Nucleolus	NA
IV	HDAC11	Primarily nucleus	NA
Other	TCF1	Nucleus	NA
Other	LEF1	Nucleus	NA

↑, upregulation; ↓, downregulated; NA, not available, Mn, manganese; BMAL1, brain and muscle arnt-like 1; iPSC, induced pluripotent stem cells, Foxo3a, Forkhead box O3; ATP5B, ATP synthase subunit β; SOD2, superoxide dismutase.

## Abbreviations

6-OHDA	6-hydroxydopamine
AA	anacardic acid ACAT1
Ac-CoA	Acetyltransferase 1
α-Syn	α-synuclein
ATP5B	ATP synthase subunit β
ATP13A2	ATPase 13A2
BMAL1	brain and muscle arnt-like 1
CS	citrate synthase
DA	dopaminergic
Foxo3a	Forkhead box O3
GCN5L1	GCN5-like 1
GLAST	glutamate aspartate transporter
GLT-1	glutamate transporter 1
GSK3β	glycogen synthase kinase 3 beta
GWASs	genome-wide association studies
H2A	Histone 2A
H2B	Histone 2B
H3	Histone 3
H4	Histone 4
HAT	histone acetyltransferase
HDAC	histone deacetylases
HIF-1α	hypoxia inducible factor 1α
IDH2	isocitrate dehydrogenase 2
iPSC	induced pluripotent stem cells
KDAC	lysine deacetylase
KAT	lysine acetyltransferase
LBs	Lewy bodies
LC3	light chain 3
LCAD	long-chain acyl-CoA dehydrogenase
L-DOPA	Levodopa
LEF1	lymphoid enhancer-binding factor 1
LONP1	Lon peptidase 1
LRKK2	leucine-rich repeat kinase 2
Mn	manganese
MPP^+^	1-methyl-4-phenylpyridinium iodide
MPTP	phenyl-1236-tetrahydropyridine
MT	microtubule
NA	not available
NaB	sodium butyrate
NAD^+^	nicotinamide adenine dinucleotide
NF-κB	nuclear factor Kappa-B
NMN	nicotinamide mononucleotide
PD	Parkinson’s disease
PGC-1α	peroxisome proliferator-activated receptor γ coactivator-1α
PINK1	PTEN-induced putative kinase 1
PTM	posttranslational modification
SAHA	hydroxamic acid
SIRT	sirtuin
SNpc	substantia nigra pars compacta
SOD2	superoxide dismutase
TCF1	T cell-specific transcription factor 1
TSA	trichostatin A
VPA	valproate
